# In Vivo and In Vitro Response to a Regenerative Dental Scaffold

**DOI:** 10.3390/ma17215384

**Published:** 2024-11-04

**Authors:** Maree L. Gould, Xiaoxuan Deng, Karl Lyons, Azam Ali

**Affiliations:** 1 Centre for Bioengineering & Nanomedicine (Dunedin), Faculty of Dentistry, Division of Sciences, University of Otago, P.O. Box 56, Dunedin 9054, New Zealand; 2 Sir John Walsh Research Institute, Faculty of Dentistry, University of Otago, P.O. Box 56, Dunedin 9054, New Zealand

**Keywords:** dental biomaterials, biocomposite, dental tissue, scaffold-based, pulp tissue regeneration

## Abstract

As dental pulp contains the stem cells necessary for regeneration, the tooth should hold the intrinsic capacity for self-repair. A triphasic hybrid dental biocomposite (3HB) composed of biocompatible biopolymers to provide strength, antibacterial properties and protein-based cell support could provide a conducive microenvironment for the regeneration of dental structures. 3HB was incorporated into Mineral Trioxide Aggregate (ProRoot MTA) to construct a malleable injectable implant. Human tooth pulp cells (hDPCs) significantly increased proliferation in the presence of 3HB+MTA compared to 3HB or MTA alone. Cell viability decreased with MTA alone but increased with 3HB and 3HB+MTA. 3HB+MTA was implanted into the residual tooth of drilled Wistar rat M2 molars for up to 45 days. Stereological analysis from micro-CT images showed the volume of the tooth remaining. Histologically, regenerative pulpal architecture was seen invading 3HB. A continuous odontoblastic profile lined a deposit of dentin-like material suggesting reparative dentinogenesis. Overall, no infection or encapsulation was seen. Immunohistochemically, odontoblasts were seen along the margins of the wounded tooth undergoing repair. Mesenchymal cells (MSCs) were seen at the base of the drilled tooth and by 21 days had translocated into the implant itself. Cells stimulating remineralization were highly expressed in the tooth undergoing repair. CD146-positive MSCs were seen in the center of the implant, possibly stimulating remineralization. In conclusion, behavior of 3HB^+^ in vitro and in vivo provided a promising start as 3HB+MTA may serve as a viable regenerative scaffold for pulp regeneration; however, this should be further studied before clinical use can be considered.

## 1. Introduction

A major goal of biomedical sciences in this century is to develop clinically relevant strategies for tissue regeneration. Emerging understanding of the interactions between biomaterials and morphogenic factors (e.g., growth regulators, genotypes, phenotypes, etc.) has accelerated translational research in the field of dental pulp tissue engineering. 

Dental caries develops when the protective enamel of the tooth is demineralized and oral bacteria penetrate the dentin exposing the dental pulp to infection and inflammation that stimulates pain. Current dental therapies focus on ablation of the disease as repairs are achieved using artificial materials that lack many of the important necessary biological characteristics of the natural tooth. Materials currently used for tooth restoration are selected based on their properties, such as biocompatibility, durability and their ability to promote healing. Such materials include amalgam, which is a mixture of mercury with silver, tin, and copper, is durable, long-lasting, and cost-effective, but is not tooth colored and does contain mercury, which has raised health concerns. Ceramics are typically made of porcelain, and although it mimics natural tooth color, it is brittle and prone to fracture if not placed properly. Glass Ionomer Cement is a combination of glass particles and an organic acid that bonds chemically to the tooth structure and releases fluoride, which can help prevent decay, but lacks mechanical strength. Zirconia is a zirconium dioxide ceramic that is extremely strong, but very expensive.

Preserving the dental pulp would directly contribute to the improvement of tooth prognosis as the pulp provides nutrition and detects pathogenic stimuli. Pulp capping materials applied to the dental pulp will protect it and promote healing when the pulp is exposed, such as in deep cavities. Materials used for pulp capping must promote tissue healing, protect the pulp and provide a good seal. Pulp capping materials include Calcium Hydroxide (Ca(OH)_2_) as a paste which promotes dentin bridge formation and has antibacterial properties but dissolves over time, leading to failure. Biodentine is a calcium silicate-based material that is biocompatible, promotes dentin formation, is easy to handle and sets faster than MTA but is still very expensive compared to traditional materials like calcium hydroxide. Resin-Modified Calcium Silicate provides a good seal, promotes dentin formation and sets faster than traditional calcium hydroxide but is not as biocompatible as pure calcium silicate materials, like MTA. An alternative or supplement to the traditional treatment of today could allow tooth regeneration while addressing the people’s needs, as well as the impact on oral health and general well-being that occurs due to dental pain, dental extractions and wearing dental prostheses to replace missing teeth.

Natural protein-based intermixed blends or composites have previously been investigated individually as promising scaffold materials for dental pulp regeneration. Natural macromolecules display the inherent ability to perform very specific chemical, mechanical or structural roles. Specifically, protein- and polysaccharide-based biomaterials have come to light as the most promising candidates for many biomedical applications due to their biocompatibility capability to function as matrices facilitating cell–cell and cell–matrix interactions. Current therapies involve the regeneration of dental pulp tissues with natural materials with proper biological functions such as scaffolds that mimic native extracellular matrix (ECM) that have pleiotropic effects, ranging from cell adhesion to cell survival. Current therapies in dental regenerative medicine tend to focus on directly introducing stem cells into damaged areas. Whilst highly debated due to safety considerations, cell transplantation therapies have been successfully implemented in the dental clinic. A recently published clinical trial showed pulp regeneration and continued root development in immature single-root permanent teeth treated with neural crest-derived mesenchymal stem cells in the form of autologous stem cells from human exfoliated deciduous teeth (SHED) [1]. Twenty six patients received SHED implantation, and although dentin regeneration was limited, all patients regenerated highly vascularized dental pulp with tooth root development in all SHED treated patients compared to the untreated controls [2]. However, there are limitations, as observed in animal studies as the transplanted cells can migrate out of the transplanted location [3] or die within the month [4,5]. 

A more promising approach may lie in attracting our own stem cells to the injury site by promoting a conducive microenvironment for cell recruitment and regeneration thereby stimulating the body’s natural healing mechanisms, possibly resulting in more effective sustainable tissue repair yielding better long-term results. Previously, we examined numerous physicochemical, mechanical and applied cell assay approaches on this material [6], but limited information is available on the regenerative potential of the 3HB biomaterial in in vivo regenerative dentistry, thus enabling a more biological approach. The naturally derived polymers included within this biomaterial facilitates cell attachment and proliferation essential for dental pulp regeneration as 3HB has properties aligned with the tissue to be regenerated. Another intent of this study was to use renewable resources, including keratin from sheep wool, chitosan from crustacean shells and collagen from tendons that are abundant in New Zealand at a low cost [7]. Although a major problem exists with amalgamating these materials due to their respective isoelectric points causing precipitation, nevertheless, intermixed blending of these biomaterials may potentially provide the ideal material for regenerative dentistry. 

The commercially available endodontic reparative cement Mineral Trioxide Aggregate (ProRoot™ MTA, Dentsply, Charlotte, NC, USA) is used as a pulp capping and pulpotomy material due to its chemical likeness to the bone, its capability of inducing stem cell osteoblastic differentiation [8,9], the promotion of bone formation by releasing calcium ions [10] and producing deposits that resemble hydroxyapatite [11,12]. MTA possesses a high alkaline pH of around 14 that provides antibacterial actions by decreasing the bacterial biofilm [13,14] but is unable to stimulate reparative dentinogenesis [15] as cells cannot survive at such a high pH. The replacement of pulp tissue with such synthetic materials without any proper biological function could lead to the failure of the restoration and consequential tooth loss. 

Utilization of tissue bioengineering would provide a clinical-grade preparation capable of tooth regeneration as an optimal treatment for preventing a tooth from becoming lost due to disease. This innovative tissue bioengineering research project incorporated bioactive molecules into a scaffold to regenerate or replace dental tissue. The aim of this in vitro and in vivo evaluative study was to determine if the incorporation of natural bioactive polymers into MTA stimulated a regenerative response by mimicking the natural ECM provoking a regenerative response of vital new tooth pulp tissue enabling the engineering of functional dental pulp and dentin tissue.

## 2. Materials and Methods

In a previous study, we developed a triphasic hybrid biomaterial (3HB) detailed in Ali et al., 2020 [6]. The major challenge that we encountered in our initial study was the amalgamation of the protein-based biopolymers within a desirable pH suitable for the tissue interaction as these proteins precipitated when moved beyond their respective isoelectric points. Strategically, optimization of protein concentrations with respect to the mineral components was overcome in the intermixed blend preparation. The final preparation of 3HB consisted of a processed blend of bioceramic and biopolymers allowing the development of a putty-type, malleable biomaterial that had a final pH of 7.7. 

### 2.1. Tooth Collection and Dental Pulp Recovery

This research was approved by the Otago University Human Ethics committee (H21/051) and in consultation with the Ngāi Tahu Research Consultation Committee. Information related to tooth collection and dental pulp tissue extraction and the human dental pulp cells (hDPCs) isolation processes has been published previously [16].

### 2.2. Cell Viability and Cytotoxicity Assay

The toxicity and biocompatibility of 3HB was characterized in vitro. Samples (100 μL) of MTA, 3HB or 3HB + MTA were applied to the well of a 48-well plate and sterilized by exposure to UV light for 20 min before rinsing with sterile PBS. The hDPC cell line had an epithelial-like morphology that grew in a monolayer attached to a substrate. Assessment of the number of viable cells of each material was performed with the LIVE/DEAD^®^ cell viability/cytotoxicity assay (L-3224, Molecular Probes, Invitrogen, Waltham, MA, USA). Briefly, an assay solution containing 4 μM calcein AM and ethidium homodimer-1 was pipetted onto each substrate and incubated at 37 °C for 10 min before visualization of fluorescence with an Olympus AX70 microscope using the Fluorescent attachment Q software. Living cells were identified due to the enzymatic conversion of calcein AM to calcein (excitation 494 nm, emission 517 nm). Dead cells were identified by the binding of ethidium homodimer-1 to the nucleic acids of cells with damaged cell membranes (excitation 528 nm, emission 617 nm). 

### 2.3. Cell Proliferation

Proliferation was assessed by a colorimetric assay using 3-(4,5-dimethylthiazol-2-yl)-5-(3-carboxymethoxyphenyl)-2-(4sulfophenyl)-2h-tetrazolium (MTS) reagent (CellTiter 96 Aqueous One Solution, Promega, Madison, New York, NY, USA). Human dental pulp cells (hDPCs) were seeded into each well at a density of 5 × 10^3^ cells/well and were grown in the presence of MTA, 3HB or 3HB+MTA in a humidified atmosphere at 37 °C and 5% CO_2_. After 24, 48 and 72 h, absorbance was recorded at 490 nm using a SmartSpec 3000 spectrophotometer (BioRad Laboratories, Hercules, CA, USA). Measurements were repeated 3–5 times on different days.

### 2.4. Surgical Procedure

Fourteen healthy young adult male Wistar rats of ~300 g in weight were used as experimental animals as they have a wider head than other rat types making the mouth more accessible. The research was approved by the Otago University Animal Ethics committee (21/41) and according to the ARRIVE guidelines. Anesthesia was induced with Ketamine (0.5 mL/kg) and Xylazine (10 mg/kg). To minimize postoperative discomfort, Carprofen, a non-steroidal anti-inflammatory analgesic (5 mg/kg), was administered subcutaneously 20 min prior to surgery and 2 days post-operatively. Amphoprim (30 mg/kg) was injected 30 min prior to surgery and post-surgery as a prophylactic antibiotic. Post anesthesia, the rats were immobilized on their abdomen, the incisors hooked over the wire. The lower teeth were stabilized, and the tongue was pulled off to one side. Lignocaine (4 mg/kg) was given as a local anesthetic. A substantial cavity was drilled completely through the randomly selected right or left M2 molar and the UV sterilized 3HB was inserted. The other side of the jaw served as a control. The animal was given a subcutaneous injection of Atipamezole (2.5 mg/kg) and allowed to recover. The animals were housed individually, and the intake of fluids and food was monitored for 45 days postoperatively. The animals were also monitored primarily for signs of pain, infection and proper activity. At 7, 14, 21 or 45 days post-implantation (n = 3,4 for each time period), the animals were sacrificed by carbon dioxide inhalation with animals selected randomly; all animals were included in the experiment. 

### 2.5. Three-Dimensional (3D) Microarchitectural Analysis of Implanted 3HB

The use of a micro-CT (μ-CT) allowed analysis of the entire 3D structure, without the need for dissection or histology. Each mandible was trimmed of soft tissue and scanned using a SkyScan μ-CT scanner (SkyScan 1172 high-resolution Microtomograph, Bruker, Antwerp, Belgium). SkyScan has an X-ray source with a focal spot of <5 μm and was operated at 40 kV, 250 μA and used a 10 Mp detector. Two-dimensional (2D) images were obtained by rotating the sample 180° at a rotation of 0.4° per sample. After completion of the 1 hr scanning process, the specimens were placed back into the formalin solution, then processed for Histology. The 2D images were compiled and stacked using ImageJ (version 1.53g US National Institutes of Health, Bethesda, MD, USA, https://imagej.net/ij/ (accessed on 29 September 2024) to generate a 3D rendering of the external surface of the root and the internal root canal. 

Stereological analyses are well-grounded mathematical methods that allow quantitative assessment of 3D structures from planar sections from the object of interest. Using the fractionator principle [17], a slice was taken every 30th section thereon to become systemic uniform samples. The tooth was identified due to the presence of enamel and the implant identified as radiopaque. The tooth volume was estimated as previously described [18]. 

### 2.6. Histology

After sacrifice, the entire mandible was excised along with surrounding tissue and fixed in 10% neutral buffered formalin for 24 h, then decalcified for histological analysis. Kidney, liver and tongue were also taken to screen for toxicity. Samples were blinded to the trained Histologist. Serial sections (4 μm) were cut perpendicularly through the implants, and the sections stained with either Hematoxylin and Eosin (H&E) for morphological analysis or toluidine blue for mast cells and visualized using light microscopy. Images were captured using Spot analysis software (Diagnostic Instruments Inc, Sterling Heights, MI, USA) on an Olympus BX-51 microscope with a Spot RT color camera attachment. 

Sections (5 µm) were deparaffinized in xylene and rehydrated with graded alcohol into distilled water. Then, either slides were stained with Hematoxylin and Eosin (H&E) or toluidine blue. Briefly, sections were stained with Harris hematoxylin for 10 min and washed thoroughly with distilled water followed by aqueous eosin staining for 2 min. Then, slides were washed and dehydrated through alcohol into xylene and mounted with DPX. Alternatively, slides were immersed in 0.5% toluidine blue working solution (pH 2.0) for 20 min before washing three times in distilled water. The sections were dehydrated by immersing the slides briefly in 100% ethanol then xylene and mounted using DPX. Toluidine blue staining results in red/purple metachromatic staining of mast cells against a pale blue orthochromatic background tissue. Toluidine blue fortuitously also stains plant tissue identifying food in between the animals teeth [19]. Using toluidine blue stained sections, the mast cell location in relation to the implant was determined. 

### 2.7. Immunohistochemistry

Sections were dewaxed in xylene, rehydrated through descending alcohol solutions, then placed in a prewarmed solution of citrate buffer (1 mmol/L, pH 6.0) and heated in a microwave oven at 90 °C for 10 min. After the immersed slides cooled to room temperature, the sections were rinsed in TRIS-buffered saline (TBS: 25 mmol/l TRIS, 0.15 mol/L sodium chloride, pH 7.6) for 10 min, followed by a 10 min wash in TBS containing 0.5% (*v*/*v*) Triton X-100 for permeabilization. 

For immunofluorescent staining, sections were incubated with 20% donkey serum (Sigma Aldrich, St Louis, MI, USA) as a block. Sections were incubated overnight at 4 °C with rabbit anti-dentin sialoprotein (DSSP) clone 2C12.3 (MABT37; Abacus, Phoenix, AZ, USA) in combination with either mouse anti-STRO-1 (ab214086; Abcam), mouse anti-CD44 (ab6124; Abcam, Cambridge, UK) or mouse anti-CD146 (ab24577; Abcam) overnight at 4 °C in TBS with 0.5% (*v*/*v*) Triton X-100, 3% (*w*/*v*) BSA. Control sections were incubated with normal rabbit serum in place of primary antiserum to indicate any non-specific staining. The next day, the slides were incubated with a combination of AlexaFluor 555 anti-mouse and AlexaFluor 488 anti-rabbit (Invitrogen) at 1:1000 for 2 h. Slides were coverslipped using Vectashield (Vector laboratories, Burlingame, CA, USA). 

For chromogenic detection, sections were incubated with 20% swine serum for 10 min. Tissue sections were subsequently incubated overnight at 4 °C with antiserum to visualize 3HB+MTA diluted at 1:100 in TBS, 0.5% (*v*/*v*) Triton X-100, 3% (*w*/*v*) BSA. Control sections were incubated with normal rabbit or mouse IgG. The next day, sections were incubated for 1 h with swine anti-rabbit or anti-mouse IgG conjugated to HRP. Sections were then washed with TBS for 20 min. Immunoreactive peptide was visualized by hydrogen peroxide (2 mg/mL) and diaminobenzidine (0.7 ng/mL) (Fast DAB, Sigma, San Diego, CA, USA) in distilled water. The sections were counterstained with Celestine blue for 10 s then coverslipped using DPX. 

### 2.8. Statistical Analysis

Statistical analysis was performed using GraphPad Prism version 9.0.2, GraphPad Software (La Jolla, CA, USA). A one-way ANOVA was used to compare total cell number, and a two-way ANOVA was used to compare cell viability with Tukey’s post hoc analysis with significance set at <0.05. 

## 3. Results

### 3.1. Cell Phenotype, Proliferation and Viability Results

**Figure 1:**
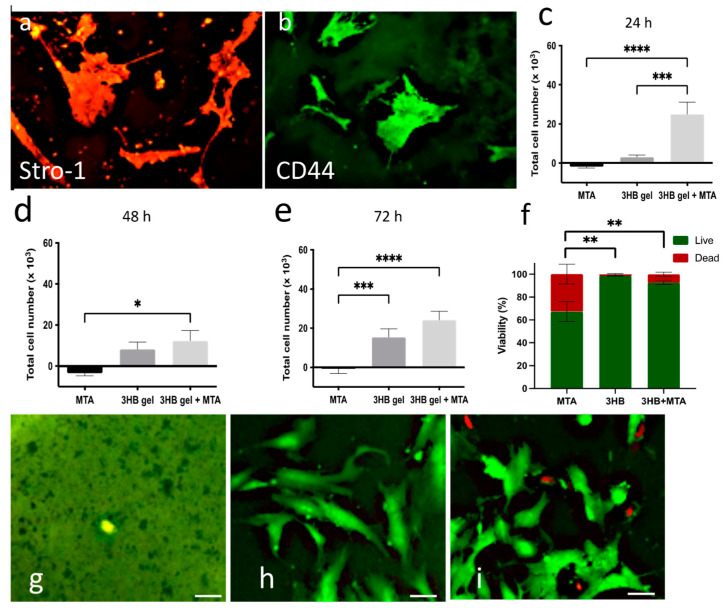
Human dental pulp cell phenotype, proliferation and viability. (Immunohistochemistry on human dental pulp cells (hDPCs) confirmed protein expression of (**a**) stro-1 (in red) and (**b**) CD44 (in green). Comparison of cell proliferation in the presence of MTA, 3HB gel and 3HB gel combined with MTA at (**c**) 24 h, (**d**) 48 h and (**e**) 72 h. (**f**) Cell viability in the presence of MTA, 3HB gel and 3HB gel combined with MTA at 72 h. Viability of hDPCs in the presence of (**g**) the dental filler MTA alone, (**h**) 3HB alone and (**i**) 3HB combined with MTA. Bar = 50 μm. Green fluorescence represents living cells, and red fluorescence indicates dead cells. Bars represent mean ± SEM. n = 3. * *p* < 0.05, ** *p* < 0.01, *** *p* < 0.001, **** *p* < 0.0001.

At all timeframes (Figure 1c–e), the presence of MTA alone significantly reduced cell proliferation. Whereas 3HB alone showed an increase in proliferation, the combined 3HB with MTA showed that the hDPC proliferation was significantly increased compared to 3HB alone (ANOVA, *p* < 0.001). The presence of MTA has been shown to promote osteogenesis [20], and this may be acting in concert with 3HB to further promote cell proliferation.

The cells expressed the mesenchymal stem cell (MSC) markers STRO-1 (Figure 1a) and CD44 (Figure 1b), indicating that these cells have the ability to self-renew, differentiate into mesenchymal and non-mesenchymal lineages and are recognized as key determinants of healing [21].

At 24 h (DFn = 3, DFd = 16, *p* = 0.0110) and 48 h (DFn = 3, DFd = 16, *p* = 0.0009), cell viability in the presence of 3HB alone and 3HB combined with MTA were significantly increased compared to MTA alone. At 72 h (Figure 1f), this effect was even more obvious (F = 21.79. DFn = 2, DFd = 12, *p* = 0.0001). Whereas the cells did not survive in MTA alone due to the high alkalinity (Figure 1g), the cell viability significantly increased in the presence of 3HB (Figure 1h) and 3HB combined with MTA (Figure 1i). 

### 3.2. Post-Surgery

**Figure 2:**
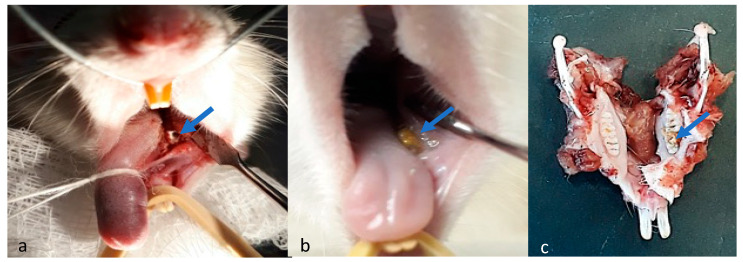
Dental Surgery. (**a**) A cavity (blue arrow) was drilled into the molar of the rat (**b**) then filled with 3HB combined with MTA. (**c**) Images of the same rat jaw after 45 days.

Some of the M2 molar of the rat was drilled away leaving a substantial cavity (Figure 2a, blue arrow) that was then filled with 3HB+MTA, capped with MTA alone and left to set (Figure 2b, blue arrow). At 7, 14, 21 and 45 days, the animals were sacrificed, and the lower jaw was removed (Figure 2c, blue arrow). Body organs that indicate toxicity, along with the tongue that sat alongside the implant, were also removed for histological analysis. 

As expected, body weight increased incrementally with age from pre-implant to 7 to 45 d (*p* < 0.0001). Animals remained healthy and consumed between 20 and 30 g of food/day and between 30 and 50 mL of water/day until sacrifice, indicating that the implant had no adverse effects on appetite, and no sign of tooth infection was seen. Liver weights increased from 18.9 ± 1.3 g at 7 days to 25.5 ± 2.3 g at 45 days. Kidney weights increased from 1.8 ± 0.04 g at 7 days to 2.5 ± 0.18 at 45 days. 

### 3.3. Stereology Results

**Figure 3:**
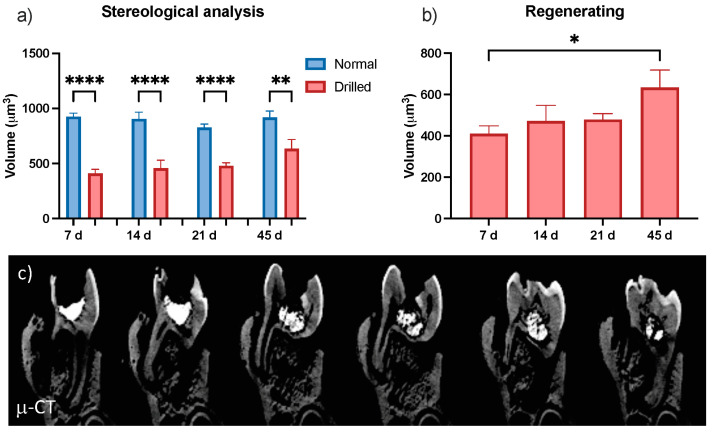
Micro-CT (μ-CT) images. (**a**) Stereological analysis of the volume of the normal and drilled tooth over time. (**b**) Volume of the regenerating tooth alone. Bar = mean ± S.E.M. n = 3, * *p* < 0.05, ** *p* < 0.01, **** *p* < 0.0001. (**c**) Reconstruction of images taken through the z-axis of the 7-day tooth using micro-CT exhibiting the radiopaque implant.

Stereological analysis showed that the tooth was mostly drilled away, with an apparent trend in recovery of the tooth volume by 45 days; in testing the regenerating (drilled) samples alone, these changes were significant (Figure 3a, *p* = 0.05). The micro-CT images (Figure 3b) provided images of the radiopaque implant at each time point.

### 3.4. Overall Histological Analysis 

**Figure 4:**
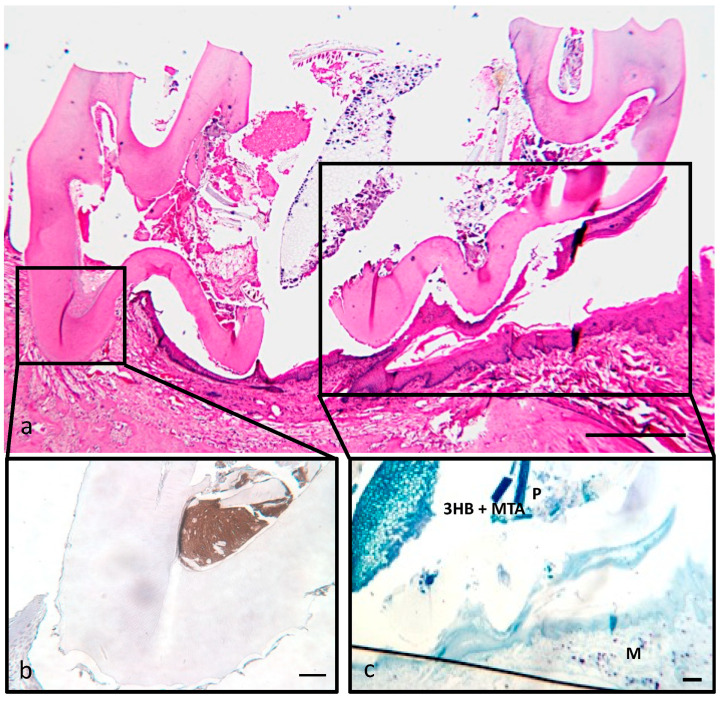
Identification of 3HB^+MTA^ and mast cell location. (**a**) Low magnification image of the implanted tooth showing most of the tooth drilled away at 7 days. (**b**) Positive immunostaining, (in brown) easily identified the implant. (**c**) Mast cells were seen deep in the gum tissue, but not in the dental pulp or around the implant (M). Bar = 1 mm.

Positive immunostaining (Figure 4b) identified the implant from the natural tooth architecture. The presence or absence of mast cells in the dental pulp are controversial in the literature. Studies using human samples have shown that mast cells are present in normal dental pulp [22] whilst others have disagreed with these findings, showing a complete absence [23], or only in inflamed tissue [24]. Furthermore, there is a lack of information regarding the presence/absence of mast cells in the dental pulp of rats [25,26]. In this study, mast cells were clearly seen in the tissue underlying the tooth (Figure 4c), but at no time were mast cells seen within the dental pulp tissue indicating that the presence of 3HB+MTA in the tooth did not instigate an inflammatory response. 

### 3.5. Histological Analysis of the Removed Organs 

Histologically, all organs sat within normal range. When examining the liver specimens, in general, normal architecture was seen. Fatty liver (5%) was seen in one animal at 45 days. A small area of infection was seen localized within one liver at 14 days. Overall, these are minor changes. 

The tongue that was adjacent to the implant was taken, the surface papillae is covered by stratified squamous epithelium that is keratinized and the ventral surface of the tongue that is protected from the harsh environment are non-keratinized. These papillae and the underlying muscle were normal in all animals. The glands (sebaceous and mucous) appeared normal. 

In the kidney, the glomerulus, tubules and ductal architecture were normal in most animals. Encapsulated renal cell carcinoma, possibly papillary cancer, with numerous small basophilic cells [27] was seen in one animal’s kidney at 45 days; this disease is five times more prevalent in males [28]. In the other kidney, loss of the bowman’s space surrounding the glomerulus was seen, and the ‘normal kidney’ tissue seemed compressed; however, the tissue weight was normal. Because of this incidental finding, other tissue types were also taken, but heart, lungs, pancreas and spleen were all normal.

### 3.6. Histological Analysis Results

**Figure 5:**
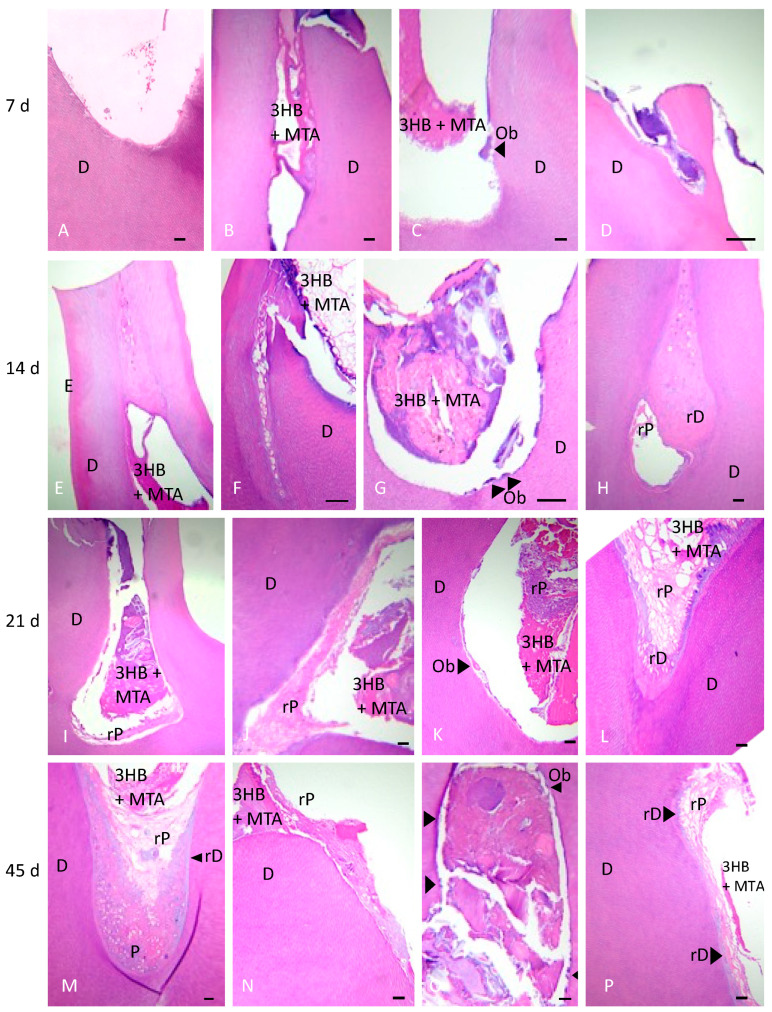
Histological aspects at 7, 14, 21 and 45 days. Hematoxylin and Eosin (**H**,**E**) sections of tooth tissue at 7 (**A**–**D**), 14 (**E**–**H**), 21 (**I**–**L**) and 45 days (**M**–**P**) are shown. Bar = 50 μm. Dentin (D), triphasic (3HB), enamel (E), osteoblasts (Ob; black arrows), regenerating dentin (rD) and regenerating pulp (rP) are marked.

Implanted 3HB appeared as a slightly basophilic material plugged into the drilled root canals. At 7 days, 3HB was visible, but little change was seen overall (Figure 5A–D). At 14 days, 3HB was still present at the partial pulpotomy site, (Figure 5E), and no infection was present where 3HB resided. Obvious infection was seen, however, where 3HB was not present or had washed away. In the presence of 3HB, (Figure 5F) some cellular infiltration was beginning to appear, (Figure 5G) odontoblasts were becoming more frequent and (Figure 5H) some root canals showed an osteodentin-like material beginning to form. By 21 days, (Figure 5I,J) regenerative pulp became more obvious, and (Figure 5K) areas suggestive of odontoblastic cell regeneration were also observed. (Figure 5L) Organized vertical parallel tissue started to form, possibly resembling a reparative dentin-like substance lining the existing dentin walls projecting into the pulp. At 45 days, (Figure 5M) 3HB was present in the pulpotomy site alongside (Figure 5N) normal pulpal tissue architecture with pulp cells invading 3HB. A continuous odontoblastic profile was seen (Figure 5O) alongside a pale basophilic heterogeneous deposition of amorphous regenerating dentin-like material (Figure 5P) suggesting reparative dentinogenesis. Furthermore, 3HB was not encapsulated by the native tissue, did not provoke an inflammatory reaction or necrosis and seemed to control infection. 

### 3.7. Immunohistochemistry Results for DSP and Stro-1

**Figure 6:**
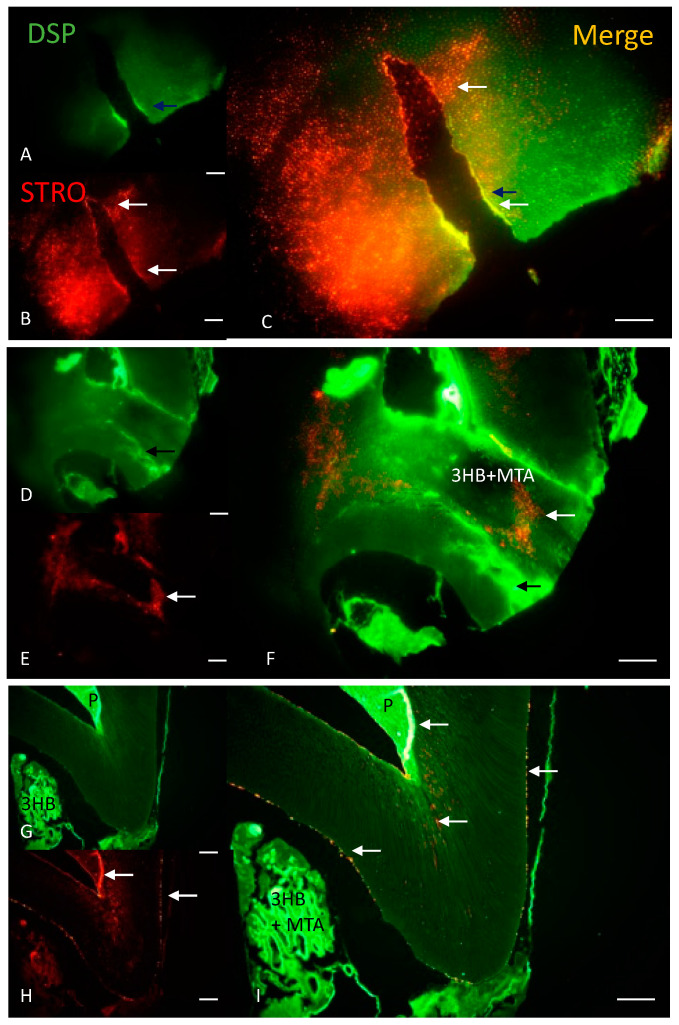
Immunohistochemistry for dentin sialoprotein and Stro-1 in the regenerating tooth. Dentin sialoprotein ((DSP) in green; **A**,**D**,**G**) and Stro-1 (in red; **B**,**E**,**H**) are shown with merged images (**C**,**F**,**I**,) at 21 (**A**–**F**) and 45 days (**G**–**I**). (**A**) Odontoblasts were seen on the upper edge (black arrow), (**B**) human mesenchymal stem/precursor cells on the upper and lower edge (white arrow) of the drilled tooth (**C**) with both cell types acting in concert. (**D**) Odontoblasts surrounded the 3HB+MTA implant (black arrow), whereas (**E**) stem cells had translocated into the implant (white arrow), (**F**) showing the different actions of the different cell types. (**G**) Odontoblasts were seen in the pulp and in 3HB+MTA, and (**H**) stem cells were seen lining the edges of the tooth (**I**) as both cell types were seen in the regenerating tooth. Bar = 100 μm.

Dentin sialoprotein (DSP) is a dentin extracellular matrix (dECM) protein that plays a critical role in odontoblast differentiation, is the predominant non-collagenous protein in dentin and is a marker of dentinogenesis [29]. In the rat tooth, particularly at 21 days rather than other time points, DSP was seen along the margins of the drilled tooth undergoing repair (Figure 6A–C). Odontoblasts were also seen within 3HB+MTA implanted into the drilled tooth (Figure 6D). By 45 days, Stro-1 was seen leading out from the pulp into the tooth and within 3HB+MTA (Figure 6G). 

Stro-1 is a marker for the mesenchymal stem cell progenitor subpopulation from primary dental pulp-derived stem cells [30]. Mesenchymal cells were seen at the base of the drilled tooth (Figure 6B) and by 21 days, had translocated into the implant itself (Figure 6E,F). At 45 days, stro-1 was seen on the margins of the regenerating tooth (Figure 6H,I). 

### 3.8. Immunohistochemistry Results for CD44 and CD146

**Figure 7:**
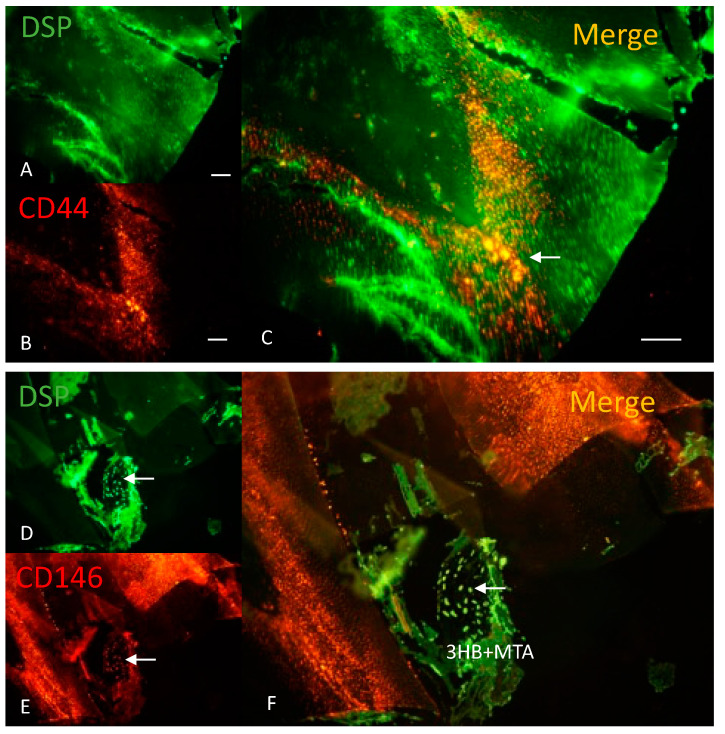
Immunohistochemistry for Dentin sialoprotein with CD44 and CD146 in the regenerating tooth. Dentin sialoprotein ((DSP) in green; **A**,**D**), CD44 (in red; **B**) and CD146 (in red; **E**) with merged images (**C**,**F**) at 21 days. (**A**) In the dentin, DSP was seen along the tooth margin, whereas (**B**) CD44 highlighted odontogenic cells with active mineral deposition in the dentin (white arrow) (**C**) as these cells acted in concert to repair the wounded tooth. (**D**) DSP staining (white arrow) combined with (**E**) CD146 staining (white arrow) (**C**) highlighted the presence of odontoblasts cells within the implant. Bar = 100 μm.

CD44 is expressed in odontogenic cells with active mineral deposition during tooth development. In the wounded tooth at 21 days (Figure 7A–C), CD44 was highly expressed in the tooth undergoing repair as these cells stimulated remineralization.

CD146 is a cell adhesion molecule and integral membrane glycoprotein at the intercellular junction and a marker for MSCs. CD146-positive MSCs possess high migration ability and were seen in the center of the implant (Figure 7D–F), possibly stimulating remineralization.

## 4. Discussion

An essential component in the regeneration of any tissue is the use of a proper supportive material to provide strength for tissue support and sites for cell adhesion, proliferation and differentiation thus promoting tissue regeneration by mimicking the characteristics of natural ECM. The aim of the current investigation was to compare the efficacy of a combination of three polymeric biomaterials into a hybrid biocomposite dental implant for the regeneration of dental pulp and dentin-like hard tissue within a biological system (in vivo, small animal model). 

Our previous trials of in vitro testing using mouse dental pulp cells (MDPC-23) grown directly on the 3HB+MTA substrate showed an increase in cell proliferation above the no treatment control showing that the substrate was non-toxic allowing the cells to adhere and proliferate with a high cell viability [6]. In this study, using human dental pulp cells (hDPCs) in vivo testing showed equal cyto-compatibility.

When tested in vivo, the formation of pulp and pulp–dentin tissue had initiated; however, overall hard tissue formation was very limited. Implanted 3HB appeared as a slightly basophilic material. Over time, the implants exhibited an abundant ingrowth of tissue, odontoblasts (as identified using immunohistochemistry) became more frequent and some root canals showed an osteodentin-like material of organized vertical parallel tissue possibly resembling a reparative dentin-like substance projecting into the pulp. A continuous odontoblastic profile was seen alongside a pale basophilic heterogeneous deposition of amorphous regenerating dentin-like material suggesting reparative dentinogenesis. 

Although these observations suggested the initial stages of hard tissue formation, similar calcification can also happen in degraded or necrotic tissues in a process named dystrophic calcification. Although these studies focused on pulp and dentin regeneration, these findings are interesting and bear further investigation in a large animal model. 

Due to the minimal size of the pulp chamber MTA sealing may not have been optimal, causing microleakage, as mild inflammation was observed in the pulp but not in the presence of the implant nor deeper in the gum as indicated by the lack of the presence of mast cells in the rat pulp. Hence, a more permanent composite bonding implant, or a more effective disinfection of the cavity to increase success of regeneration, will be required in the future.

The utmost reasons to develop pulp–dentin regeneration technology are to reinstate tooth functionality and avoid tooth loss. De novo pulp regeneration of dentin-like tissue deposition on canal walls has been achieved via stem cell-based approaches; however, transplanting exogenous cells does not commit cell lineage differentiation to odontoblasts. No cell-free approach has demonstrated dental tissue regeneration where the pulp tissue was completely removed [31,32]. 

We showed orthotopic pulp regeneration along with newly deposited dentin similar to tertiary or reparative dentin along the canal walls. Furthermore, odontoblast-like cells were seen to emerge on the existing dentinal wall; this has also been seen by other researchers [33,34]. The TGF-β family of growth factors and bone morphogenetic proteins are responsible for primary odontoblast differentiation during tooth development; however, odontoblast differentiation from dental pulp stem cells during pulp regeneration activates a different pathway where the matrix metalloprotease (MMP) family is up-regulated with Wnt/β-catenin pathway activation in the pulp cells underneath the irritated dentin [35]. 

The formation of fully operational tertiary dentin after pulp capping is the penultimate of the pulp-engineering process; however in this study, the tertiary dentin lacked organized dentinal tubules. Therefore, the quality of the regenerated pulp–dentin complex also requires further investigation. 

Odontoblast specific cells expressing DSSP lined the regenerating tooth suggesting the odontoblast phenotype of these cells [29], CD44 is expressed in odontogenic cells undergoing active mineral deposition [36] and the presence of Stro-1 and CD146 positive human dental pulp stem cells defining the stem cell niche were all detected in the regenerating pulp. These markers identify an odontoblast lineage as the right signals to allow DPSCs differentiating into odontoblast lineages appears of critical importance. 

Other studies utilized heterogeneous populations of implanted dental pulp stem cells isolated from mini-swine implanted with either a hydroxyapatite-tricalcium phosphate (HA/TCP) or a tooth fragment model when implanted in vivo which showed similar results with osteodentin depositing along the canal walls alongside regeneration of vascularized pulp-like tissue [37]. Alternatively, other researchers have observed an overproduction of regenerating dentin; for example, heterogeneous DPSCs from the canine tended to overproduce osteodentin which completely occluded the pulp space promoting calcification, but this is considered reparative rather than regenerative repair [34]. A rigid 3D printed scaffold of polycaprolactone (PCL) and hydroxyapatite nanoparticles (nHA) alginate, and collagen bioink containing hDPCs in a rat model showed a low inflammatory response and successful tissue integration. Although mechanically strong, PCL also has limitations of low cell adhesion and proliferation, and a slow degradation rate owing to its high crystallinity and hydrophobic nature as an ideal implant for tissue regeneration has yet to be definitively established, as each material exhibits unique advantages and limitations [38]. 

Type I collagen is the predominant component of the dentin matrix and dental pulp. Native collagen fibers can catalyze calcium phosphate crystallization from physiological concentrations of calcium and phosphate ions. Type I collagen in dentin presents initiation sites for calcification, even though collagen alone does not generate mineralization [39]. Therefore, collagen has been used as a capping material for regeneration of the dentin–pulp complex; however, the healing effect of collagen was prone to inflammation and infection, and hence, insufficient regeneration of dentin-like tissue was induced [40,41,42]. 

Other researchers have used a variety of methods to improve bone regeneration, such as the treatment of rats with critical size skull defects using bovine bone grafts with low level laser therapy (LLLT). The outcomes were measured using a multimodal optical coherence tomography (OCT) system to assess de novo bone formation. Bovine bone graft when combined with LLLT had the highest rate of regeneration highlighting the potential of LLLT in enhancing bone healing [43].

Other light therapy techniques that have been used to treat periodontal oral disease include the biomodulatory effects of low-dose blue light. The photobiomodulation (PBM) of gingival fibroblast cells irradiated with 400 nm light showed increased inflammatory markers in these cells. Comparatively, when the cells were first exposed to a bacterial stimuli then exposed to blue light, decreases in the proinflammatory markers IL-8 and ROS were seen indicating that PBM could modulate the oral inflammation associated with periodontitis [44].

Although 3HB + MTA provided encouraging results, there were notable limitations, particularly the low amount of hard tissue formation. These limitations suggest that further research (perhaps utilizing organoid models) and optimization of this product is needed to enhance the regenerative capabilities of these materials.

## 5. Conclusions

Triphasic 3HB combined with a dental filler (MTA) supported the attachment, growth and differentiation of human dental pulp cells confirming biocompatibility. The in vivo analysis illustrated that the implant (scaffold) supported pulp tissue ingrowth and a dentin-like material was formed. In conclusion, the behavior of this implant should be further studied in vivo, in a large animal model before dental tissue regeneration in humans can be considered, but this is a promising start.

## Data Availability

The original contributions presented in the study are included in the article, further inquiries can be directed to the corresponding author.
